# *Monascus sanguineus* May Be a Natural Nothospecies

**DOI:** 10.3389/fmicb.2020.614910

**Published:** 2020-12-22

**Authors:** Yatao He, Junlin Liu, Qian Chen, Senning Gan, Ting Sun, Shengdong Huo

**Affiliations:** ^1^College of Life Science and Engineering, Northwest Minzu University, Lanzhou, China; ^2^Center for Life Sciences, School of Life Sciences, Yunnan University, Kunming, China

**Keywords:** *Monascus sanguineus*, phylogeny, morphology, nothospecies, fungi, natural pigment, industrial strain

## Abstract

The genus *Monascus* has important economic and ecological values. In 2016, we isolated a strain *M. sanguineus*. After studying the phylogenetic relationship of *Monascus*, we believe that *M. sanguineus* is an independent species and speculate that it is a natural nothospecies. Recently, the morphological characteristics and sequences of seven genes (ITS, LSU, β-tubulin, calmodulin, RNA polymerase II subunit, β-ketoacyl synthase, and mating-type locus 1-1) of 15 *Monascus* strains were analyzed, including sequencing of multiple clones of five protein genes in four *M. sanguineus* strains. Two types of haplotypes (A and B) were observed in the five protein genes of *M. sanguineus*. Haplotype A was closely related to *M. ruber*, and haplotype B may be derived from an unknown *Monascus* species. The results demonstrated that *M. sanguineus* including type strains may be a natural nothospecies. This study laid the foundation for further exploration of the *M. sanguineus* genome, and the study may be of significant importance for the *Monascus* fermentation industry.

## Introduction

*Monascus* spp. are filamentous fungi first described by a French scientist Van Tieghem (1884). Having medicinal as well as edible uses, *Monascus* has been used in China for nearly 2000 years. Its use can be dated back to the Han dynasty (BC 202–AD 220). As a characteristic species, *Monascus*-fermented rice (also known as red yeast rice, a rice-based fermentation product) is widely consumed throughout East Asia and has a profound impact on local life and culture. *Monascus* has received worldwide attention because of its diverse products and rich beneficial metabolites. Its distribution ranges from the Korean Peninsula to the Malay Archipelago, and it is even spread globally (Lee and Pan, 2011, 2012; Shi and Pan, 2012; Chen et al., 2015). In industrial production, *Monascus* has three principal applications or products: starter culture (as a starter in various food fermentations), *Monascus*-fermented rice (as a food supplement), and functional food, which are widely used in brewing, food coloring, and healthcare industries. *Monascus* is an important source of numerous types of hydrolytic enzymes required in fermentation of foods (including red rice wine, red rice vinegar, Chinese spirits, fish paste, and fermented tofu). The beneficial secondary metabolites produced by *Monascus* mainly include *Monascus* pigments (food colorants and condiments), biofunctional components (including monacolins, γ-amino butyric acid, and dimerumic acid) (Feng et al., 2012; Hsu and Pan, 2012; Vendruscolo et al., 2014; Mérillon and Ramawat, 2017), and citrinin (safety disputed because of renal toxicity) (Kim et al., 2010; Li et al., 2012, 2015). More than one billion people have been estimated to eat *Monascus*-fermented products daily, with the most popular product being *Monascus*-fermented rice (Yang et al., 2015). Additionally, researchers have found that *Monascus* has a mutually beneficial symbiotic relationship with some bees, indicating its important ecological value (Menezes et al., 2015; Barbosa et al., 2017).

The genus *Monascus* belongs to the family Aspergillaceae and order Eurotiales. It contains 36 species; however, many of them are considered synonymous (Shao et al., 2011, 2014). Based on the study by Barbosa et al. (2017), we preliminarily unified the phylogenetic relationships of the species within the genus *Monascus* and confirmed that *Monascus* includes the *Rubri* and *Floridani* sections. The *Rubri* section consists of three species and three varieties; the *Floridani* section consists of seven species, and additionally, one species speculated to be a natural nothospecies was found (He et al., 2018). In this study, to improve the phylogenetic relationship within *Monascus* and identify the parents of suspected nothospecies, we analyzed the sequences of the internal transcribed spacer (ITS), large subunit (LSU), beta-tubulin (*BenA*), calmodulin (*CaM*), RNA polymerase II subunit (*RPB2*), beta-ketoacyl synthase (*pksKS*), and mating-type locus 1-1 (*MAT1-1*) in 15 strains of *Monascus*. Based on a polyphasic approach combining sequence data with macroscopic and microscopic characters, it is speculated that *M. sanguineus* may be a natural nothospecies; *M. ruber* is one of its parents, and the other parent may be an unknown species.

## Materials and Methods

### Strains

The strain *M. sanguineus* (CGMCC 3.19000 = RJL03) was isolated from the medicinal plant *Rehmannia glutinosa*. *M. purpureus* (Han01) was isolated from the commercially available Fujian (China) red yeast rice. The strain *M. sanguineus* (SICC 3.292) was purchased from the Sichuan Center of Industrial Culture Collection (SICC,^1^, China), and the remaining 12 *Monascus* strains were purchased from the China General Microbiological Culture Collection Center (CGMCC,^2^, China).

### Cultivation and Morphological Analyses

For observing the colonial morphology, the 15 strains (Table 1) were cultured in three points on potato dextrose agar (PDA), malt extract agar (MEA), and Czapek yeast extract agar (CYA) plates at 30°C for 7 days. Macroscopic characteristics such as soluble pigments, color of the mycelium, and obverse and reverse colony colors were studied. Single colony diameters were measured after incubation for 7 days, and the average growth rate was calculated. The hyphae were observed by the transparent tape method using an optical microscope. A drop of lactophenol cotton blue stain was placed on a glass slide. The hyphae were adhered to a transparent tape. Ethanol was added dropwise to the surface of the hyphae, and they were placed on the slide with the drop of stain. The stain solution was added dropwise on the surface of the tape, which was later covered with a coverslip. Scanning electron microscopy revealed that the strains were cultured by insert coverslip. After the hyphae were climbed, the coverslips were gently removed for treatment. The coverslips with hyphae were fixed in 2.5% glutaraldehyde for more than 4 h and further rinsed 3 times with phosphate buffer. The coverslips with samples were dehydrated with graded concentrations of ethanol (50, 70, 80, 90, 95, and 100%) for 20 min for each concentration, transferred to pure isoamyl acetate for 1 h, and coated with gold–palladium. After pretreatment of the samples, morphological characteristics such as size, shape, and pigmentation of conidia, conidiophores, ascomata, asci, and ascospores were observed under the BME Biooptical microscope (Shanghai Leica Microsystems Co., Ltd., China) and SM-5600LV low vacuum scanning electron microscope (Japan Electronic Co., Ltd., Japan).

**TABLE 1:** Strains used in this study.

**Species**	**Strain numbers**	**Substrate**	**Location and date**
*Monascus ruber*	CBS 135.60 = CGMCC 3.4701 ^NT^	Soil	India, 1884
*M. ruber*	CGMCC 3.2093	Fermented grain	Guizhou, China, 1961
*M. ruber*	FWB13	Red yeast rice	Fujian, China, 2015
*M. ruber var. albidulus*	CGMCC 3.568 ^T^	Fermented wheat grain	Liaoning, China, 1952
*M. purpureus*	CBS 109.07 = CGMCC 3.5833 ^T^	Fermented rice grain	Indonesia, 1895
*M. purpureus*	YY-1	Food coloring Commercial strain	China, Unknown
*M. purpureus*	Han01	Red yeast rice	Fujian, China, 2018
*M. purpureus var. rutilus*	CGMCC 3.2636 ^T^	Fermented grain	Fujian, China, 1961
*M. purpureus var. aurantiacus*	CGMCC 3.4384 ^T^	Fermented grain	Anhui, China, 1980
*M. sanguineus*	IMI 356821 = CGMCC 3.5845 ^T^	River sediment	Iraq, 1995
*M. sanguineus*	SICC 3.292	Fermented grain	Sichuan, China, 1960
*M. sanguineus*	CGMCC 3.2848	Fermented grain	Guangdong, China, 1970
*M. sanguineus*	CGMCC 3.19000 = RJL03	Tuber of *Rehmannia glutinosa*	Henan, China, 2016
*M. floridanus*	CBS 142228 = CGMCC 3.5843 ^T^	Sand pine roots	United States, 1987
*M. pallens*	CBS 142229 = CGMCC 3.5844 ^T^	River sediment	Iraq, 1995
*M. lunisporas*	CBS 142230 = CGMCC 3.7951 ^T^	Moldy feed for race horses	Japan, 1998
*M. argentinensis*	CBS 109402 = CGMCC 3.7882 ^T^	Soil	Argentina, 2004
*M. mellicola*	CBS 142364 ^T^	Honey of *Melipona scutellaris*	Brazil, 2017
*M. recifensis*	CBS 142365 ^T^	Pollen of *Melipona scutellaris*	Brazil, 2017
*M. flavipigmentosum*	CBS 142366 ^T^	Inside nest of *Melipona scutellaris*	Brazil, 2017
*Penicillium eremophilus*	CBS 123361 ^T^	Moldy prunes	Australia, 1988
*P. verrucosum*	CBS 603.74 ^T^	Unknown source	Belgium, 1901
*P. polonicum*	CBS 222.28 ^T^	Soil	Poland, 1927

*T, type strain; NT, neotype strain; CBS, Culture collection of the Westerdijk Fungal Biodiversity Institute (formerly known as Centraalbureau voor Schimmelcultures), The Netherlands; CGMCC (AS), China General Microbiological Culture Collection Center (formerly known as Chinese Academy of Sciences), China; SICC, Sichuan Center of Industrial Culture Collection, China; IMI, International Mycological Institute, United Kingdom.*

### DNA Extraction, Amplification, Cloning, and Sequencing

Strains were grown on MEA for 7–14 days prior to DNA extraction. Genomic DNA was extracted using Fungi Genomic DNA Extraction Kit (Beijing Solarbio Science and Technology Co., Ltd., China) as per the manufacturer’s instructions. DNA was amplified through polymerase chain reaction (PCR) using seven pairs of primers for seven genes (Supplementary Table 1). According to our study, cloning of PCR products was not required except for five protein genes in four *M. sanguineus* strains. At least 10 clones were randomly selected for each sample using the blue–white selection system, and both regular and clones were sequenced (Sangon Biotech Co., Ltd., Shanghai, China).

### Sequence Alignment and Phylogenetic Analyses

Contings were assembled using the forward and reverse sequences with the SeqMan v.7.1.0. Analysis of homology of amplified products was studied using Blastn. Further, the sequences generated in this study were submitted to GenBank via the web tool BankIt or Sequin program. Sequence datasets were generated by combining the 197 newly generated sequences and 44 sequences that we deposited from GenBank (Supplementary Table 2). Sequence alignments were performed in MAFFT^3^ and were manually optimized using MEGA 7. The best substitution model for each partition was inferred with the program MrModeltest 2.3. Phylogenetic trees were constructed through maximum likelihood (ML) analysis in raxmlGUI 1.5 using the GTRGAMMA substitution model and 1000 bootstrap replicates. Bayesian inference (BI) in MrBayes v.3.2.1 was performed using the Markov Chain Monte Carlo (MCMC) algorithm. Sequence format conversion was performed using Mesquite 3.10. Individual alignments were concatenated using Sequence Matrix v.1.7.8 for multilocus phylogenetic analyses. Each gene was analyzed separately, and further, two sequences with the highest rate of the two haplotypes were selected. Data partitioning was performed to construct a multigene phylogenetic tree.

## Results and Discussion

### Evidence for *M. sanguineus* as a Natural Nothospecies

**FIGURE 1:**
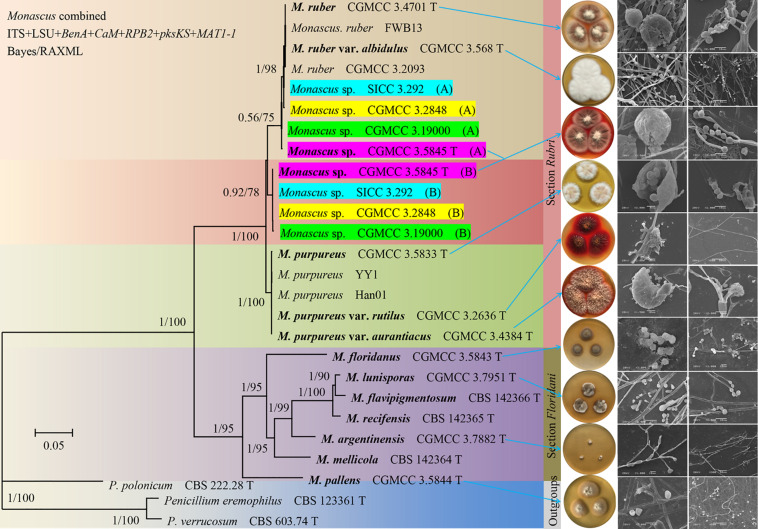
Phylogeny of *Monascus*. Phylogenetic tree constructed from Bayesian analysis of the concatenated sequences of five protein genes (*BenA*, *CaM*, *RPB2*, *pksKS*, and *MAT1-1*) and two ribosomal RNA genes (ITS and LSU) with a total length of 6,983 bp. Posterior probabilities from Bayesian analysis/bootstrap percentages from 1,000 replicates of maximum likelihood analysis are shown on major branches. The right side of the figure is the cultural characters and scanning electron microscope structural of the strains indicated by the arrow. For the sake of illustration, the *M.* sp. CGMCC 3. 5845 (A) in the figure are manually supplemented.

Our analysis revealed two well-supported sections (*Rubri* and *Floridani*) in *Monascus*. Seven lineages are present in the section *Floridani* and these lineages are treated as separate species. *M. purpureus*, *M. sanguineus*, and *M. ruber* are located in the section *Rubri*. Also, the results of this study demonstrated that four *M. sanguineus* strains including the type strain may be natural nothospecies (not found in previous research data). Additionally, two types of haplotypes (A and B) were found after cloning and sequencing of five protein genes. Haplotype A was closely related to *M. ruber*. Haplotype B may be derived from an unknown *Monascus* species (Figure 1 and Supplementary Figure 1). Haplotype B had a much higher red-pigment-producing ability than its suspected parent *M. ruber*, but its growth rate was lower than that of *M. ruber*. Thus, it was speculated that the yet unknown parent (*Monascus* sp.) of haplotype B confers the ability to produce red pigment. Additionally, the heterozygosity of the four *M. sanguineus* strains was notably different. For example, we had not cloned haplotype A of the strain CGMCC 3.5845; all SNPs were only observed at the corresponding sites in the direct sequencing of the five protein genes, and the two types of haplotypes between each hybrid strain were not completely consistent. For example, the results of cloning and sequencing demonstrated that the β-tubulin (*BenA*) gene of strain CGMCC 3.2848 had five haplotypes A and five haplotypes B (Supplementary Figure 2). For better understanding, an example of yeast (*Saccharomyces*) can be considered, which is similar to the *M. sanguineus* hybrid and has been studied in detail. The allopolyploid hybrid *S. pastorianus* was once considered the synonym of its parent strain *S. cerevisiae*; however, the parent strain *S. eubayanus*, with its most important low-temperature fermentation characteristics, was discovered after a long period (Bing et al., 2014; Wendland, 2014). Simple tests, such as DAPI staining to examine karyotypes and qPCR to assess fold changes in gene copy number, can be used to analyze the difference between chromosome ploidy in *M. sanguineus* and *M. ruber* (Waalwijk et al., 2018). It is speculated that in the previous study, the ribosomal ITS and LSU gene sequences could not distinguish between the hybrids *M. ruber* and *Monascus* sp., probably because in the chromosome of the hybrid, the chromosome containing the ribosomal RNA gene (rDNA) cluster from the *M. ruber* parent was substantially lost or reduced in length after hybridization. Referring to this example, to reflect the characteristics of *M. sanguineus* as a hybrid, we describe it as an independent species (*M. sanguineus*). The official DNA barcode for the fungal ITS region can recognize all *Monascus* species (including *M. purpureus* and *M. sanguineus*), but larger sequence variations can be observed in the *BenA* gene. Therefore, we suggest that the *BenA* gene can be used as a secondary barcode for the identification of *Monascus* species, besides the methods based on morphological features (Barbosa et al., 2017).

Natural hybridization is very common in flowering plants. Approximately 25% of plant species are reported to hybridize with other species (Arnold, 1992; Mallet, 2005). Natural hybridization plays an important role in speciation, genetic exchange, and adaptive evolution. However, it can also lead to the extinction of rare species or generate super invasive species (Hegarty and Hiscock, 2007; Zhuang et al., 2019). *M. sanguineus* (CGMCC 3.5845) was first isolated from the sediments of the Arab River in Iraq in 1995 (Cannon et al., 1995) and was later reported in an Indian plant *Punica granatum* (Rashmi and Padmavathi, 2014). The SICC 3.292 strain was isolated from fermented grain in Sichuan Province in 1960, and the RJL03 strain was isolated from the plant *Rehmannia glutinosa* in our laboratory (He et al., 2019). Four *M. sanguineus* strains from different sources used in this study showed hybridization, indicating that hybrids may not have occurred by artificial breeding but by natural hybridization. However, the origin of its formation still needs to be studied. *M. sanguineus* was considered to be the synonym species of *M. purpureus*. This study demonstrated that *M. ruber* may be one of its parents, and there are some significant differences among the four *M. sanguineus* strains. We compared the morphological characteristics of two different *M. sanguineus* strains (CGMCC 3.5845 and CGMCC 3.2848) with *M. ruber* (CGMCC 3.4701) and *M. purpureus* (CGMCC 3.5833); the results showed that the morphological structure of the ascomata, ascospores, and conidium of the two hybrid strains was different from that of *M. ruber* and *M. purpureus*, but it was close to that of *M. ruber* (Table 2). Additionally, a significant difference was observed between the two hybrids; the three *M. sanguineus* strains grew faster than the type strain CGMCC 3.5845 (Supplementary Figures 3, 4).

**TABLE 2:** Morphological characteristics of some tested strains of *Monascus*.

**Strain**	**Ascomata (μ m)**	**Ascospore (μ m)**	**Conidium (μ m)**		**Mycelium (μ m)**	**Growth rate (mm/d)**
CGMCC 3.4701	Hyaline or orange 25–40	Ellipsoidal 4.5–5.5 × 5.5–7	Rough reticulate, globose to obpyriform 8–14 × 10–18	Single or up to 9 conidia in chain	Hyaline or light brown, Oleous 3–6	4.66
CGMCC 3.5833	Hyaline or reddish 25–65	Ellipsoidal 4–5.5 × 5–7	Smooth, globose to obpyriform 8–10 × 10–12	Single or up to 5 conidia in chain	Hyaline or light brown, Oleous 3–5	3.07
CGMCC 3.5845	Brown 32–60	Ellipsoidal 6–8 × 5.5	Rough reticulate, globose to obpyriform 10–14 × 8–10	Single or up to 10 conidia in chain	Hyaline or reddish, no oleous 3–4	2.80
CGMCC 3.2848	Brown or reddish 32–70	Ellipsoidal 6–7.5 × 4–5	Rough reticulate, globose to obpyriform 8–16.5 × 7.5–14	Single or up to 10 conidia in chain	Hyaline or reddish, no oleous 3–6	4.56

### Urgent Need to Study the *M. sanguineus* Genome

With the sequencing of the *M. pilosus* genome in 2004, the study on *Monascus* has entered the era of genomics (data not shown). Genomic sequencing of *M. ruber* (M7, NRRL 1597) and *M. purpureus* (NRRL 1596, YY-1) was completed. The average genome size was found to be 24.04 Mb, containing 7 or 8 chromosomes. The sketch genome coverage rate was approximately 95.6%. This gave us a deeper understanding of the physiology of *Monascus* and revealed new methods for strain improvement (Chen et al., 2015; Yang et al., 2015; Ding et al., 2016). However, only a few genes have been sequenced in *M. sanguineus*, and characteristics of the whole genome (genome size, chromosome number, and precise structure) remain undiscovered. We suggest that first, the genome of *M. sanguineus* should be sequenced, its ploidy should be determined, and comparative genomics studies should be conducted to compare its genomic sequence with that of other *Monascus* species (especially *M. ruber* and *M. purpureus*). Further, the structural features of two subgroups of hybrids (A and B subgenomes), the homology of the *M. sanguineus* subgroup and *M. sanguineus* should be analyzed, and it should be determined whether *M. ruber* is the ancestor of the *M. sanguineus* subgroup A while investigating the donor parent of subgroup B. In addition to its economic value, *Monascus* genomics research plays an important role in recognizing structural variability, integrating phenotype–genotype association, understanding the origin and evolution of the *Monascus* genome, and elucidating the genetic structure of some important traits. A comparative genomic study of *Monascus* species will enrich the knowledge about *Monascus* genetics and biology. Additionally, as an industrial strain, *Saccharomyces* has been studied in more detail; hence, the related research cases can be assessed in the study of *Monascus* genomics.

*Monascus* fermentation and its applications are the driving forces for research on *Monascus*. This study demonstrated that *M. sanguineus* may be a natural hybrid; we suggest to describe it as an independent species, and sequence analysis of its whole genome should be performed. In recent years, to analyze the regulation of secondary metabolites in *Monascus*, molecular biological studies such as those related to genes (clusters) of the main secondary metabolites of *Monascus*, biosynthetic pathways, and regulatory mechanism have made significant progress (Chen et al., 2017). At present, we can improve the production of beneficial metabolites of *Monascus* through strain selection, optimization of fermentation conditions, and genetic modification and effectively eliminate or reduce the content of citrinin in *Monascus* products. The completion of *Monascus* genome sequencing will greatly promote the studies on various aspects of *Monascus*, by which we can better understand the fermentation characteristics of different strains and molecular mechanisms underlying metabolite production and clarify the breeding and genetic transformation of *Monascus* strains. This will be beneficial for the *Monascus* fermentation industry. During the era of molecular biology, the aim was to establish the connection between *Monascus* genetics and biological performance. The studies on *Monascus* genomics will eventually boost *Monascus* research to a global level with clear goals. In the foreseeable future, the combination of genomics and molecular biology techniques will play a major role in *Monascus* research (Chen et al., 2015).

## Data Availability Statement

The datasets presented in this study can be found in online repositories. The names of the repository/repositories and accession number(s) can be found in the article/Supplementary Material.

## Author Contributions

YH and JL conceived and designed the experiment. YH, QC, SG, and TS carried out the experiment and performed the analysis. YH wrote the manuscript. JL and SH revised the manuscript. All authors discussed the results and commented on the manuscript.

## Conflict of Interest

The authors declare that the research was conducted in the absence of any commercial or financial relationships that could be construed as a potential conflict of interest.

## Footnotes

1 http://www.sc-sicc.org.cn/

2 http://www.cgmcc.net/

3 https://mafft.cbrc.jp/alignment/server/

## Supplementary Material

Supplementary Figure 1 Single gene phylogenetic trees of the ITS, LSU, *BenA*, *CaM*, *RPB2*, *pksKS*, and *MAT1-1* gene regions of species from *Monascus*.

Supplementary Figure 2 Two haplotypes of the *BenA* gene of four *M. sanguineus* strains (only inconsistent sequences are retained after alignment).

Supplementary Figure 3 Cultural characters of 15 *Monascus* strains cultured on MEA, PDA, and CYA media at 30°C for 7 days.

Supplementary Figure 4 Mean growth rates (mm/d) of 15 *Monascus* strains cultured on PDA, MEA and CYA media at 30°C for 7 days.

Supplementary Table 1 Genes sequenced and primers for PCR.

Supplementary Table 2 GenBank accession numbers of sequences used in the molecular study.
