# Supplementary material for: Monascus sanguineus May Be a Natural Nothospecies
Source: Front Microbiol. 2020 Dec 22;11:614910. doi: 10.3389/fmicb.2020.614910 (PMC7782312; doi:10.3389/fmicb.2020.614910)
Supplement: Supplementary Figure 1 — Single gene phylogenetic trees of the ITS, LSU, BenA, CaM, RPB2, pksKS, and MAT1-1 gene regions of species from Monascus. [file Data_Sheet_1.PDF]

Supplementary Material

Table S1. Genes sequenced and primers for PCR.

Locus	Gene product	Primer(5'-3')		Size (bp)	Annealing Temperature (°C)	Extension time (s)
ITS	Internal Transcribed Spacer	V9G	TTACGTCCCTGCCCTTTGTA	967-1386	52	80
		LS266	GCATTCCCAAACAACTCGACTC			
LSU	Large SubUnit	LR0R	GTACCCGCTGAACTTAAGC	943	48	75
		LR5	ATCCTGAGGGAAACTTC			
BenA	Beta tubulin II	Bt2a	GGTAACCAAATCGGTGCTGCTTTC	549-577	56	40
		Bt2b	ACCCTCAGTGTAGTGACCCTTGGC			
CaM	Calmodulin	Cmd5	CCGAGTACAAGGAGGCCTTC	559-636	56	40
		Cmd6	CCGATGGAGGTCATGACGTGG			
RPB2	RNA polymerase II subunit	5F_Eur	GACGACCGGGATCATTTCGG	1182	52	90
		7CR_Eur	CCCATAGCTTGTTTACCCAT			
pksKS	Beta-ketoacyl synthase	pks1	TCCTTCACTTCTGCGAGGACT	1520	51	100
		pks2	AAAAGTTCTTCGCCGAGACTGT			
MAT1-1	Mating-type locus 1-1	M3F	ACAAGGTTGGACAGTAGGT	992	48	75
		M3R	TTTTCAGTCGAGATGGCTA			

Table S2. GenBank accession numbers of sequences used in the molecular study.

Strain numbers	Strain code	GenBank accession numbers						
		ITS	LSU	*BenA*	*CaM*	*RPB2*	*pksKS*	*MAT1-1*
**CGMCC 3.4701**	RUB4701N	MN156541	MN153459	MN229573	MN229607	MN229643	MN229684	MN229711
**CGMCC 3.2093**	RUB2093N	MN156542	MN153460	MN229574	MN229608	MN229644	MN229685	MN229712
**FWB13** [1]	RUB0019N	PSNO01000019	PSNO01000024	PSNO01000001	PSNO01000005	PSNO01000003	PSNO01000010	PSNO01000006
**CGMCC 3.568**	RUB0568N	MN156543	MN153461	MN229575	MN229609	MN229645	MN229686	MN229713
**CGMCC 3.5833**	PUR5833N	MN156544	MN153462	MN229576	MN229610	MN229646	MN229687	MN229714
**YY-1** [1]	PUR0003N	QDGY01000003	QDGY01000003	QDGY01000001	QDGY01000002	QDGY01000006	QDGY01000005	QDGY01000007
**Han01**	PUR0001N	MN156545	MN153463	MN229577	MN229611	MN229647	MN229688	MN229715
**CGMCC 3.2636**	PUR2636N	MN156546	MN153464	MN229578	MN229612	MN229648	MN229689	MN229716
**CGMCC 3.4384**	PUR4384N	MN156547	MN153465	MN229579	MN229613	MN229649	MN229690	MN229717
**CGMCC 3.5845**	SAN5845N/A/B	MN156548	MN153466	MN229580-83	MN229614-18	MN229650-56	MN229691-95	MN229718-19
**SICC 3.292**	SAN3292N/A/B	MN156549	MN153467	MN229584-89	MN229619-27	MN229657-63	MN229696-02	MN229720-26
**CGMCC 3.2848**	SAN2848N/A/B	MN156550	MN153468	MN229590-99	MN229628-34	MN229664-71	MN229703-06	MN229727-34
**CGMCC 3.19000**	SAN1900N/A/B	MN156551	MN153469	MN229600-02	MN229635-38	MN229672-79	MN229707-10	MN229735-39
**CGMCC 3.5843**	FLO5843N	MN156552	MN153470	MN229603	MN229639	MN229680	n/a	n/a
**CGMCC 3.5844**	PAL5844N	MN156553	MN153471	MN229604	MN229640	MN229681	n/a	n/a
**CGMCC 3.7951**	LUN7951N	MN156554	MN153472	MN229605	MN229641	MN229682	n/a	n/a
**CGMCC 3.7882**	ARG7882N	MN156555	MN153473	MN229606	MN229642	MN229683	n/a	n/a
**CBS 142364**	MEL2364N	KY511726	KY511756	KY611943	KY709143	KY611904	n/a	n/a
**CBS 142365**	REC2365N	KY511740	KY511770	KY611957	KY709157	KY611918	n/a	n/a
**CBS 142366**	FLA2366N	KY511751	KY511781	KY611968	KY709168	KY611929	n/a	n/a
**CBS 222.28**	PEN3338N	AF033475	JN939272	JN985417	AF001206	KU896848	n/a	n/a
**CBS 603.74**	PEN0374N	AB479317	AB479285	JN121539	AF001205	DQ911138	n/a	n/a
**CBS 123361**	PEN0995N	GU733341	KY645973	KY611970	KY709170	KY611931	n/a	n/a

[1] Sequence from genome sequenced strain; n/a: no sequence available.

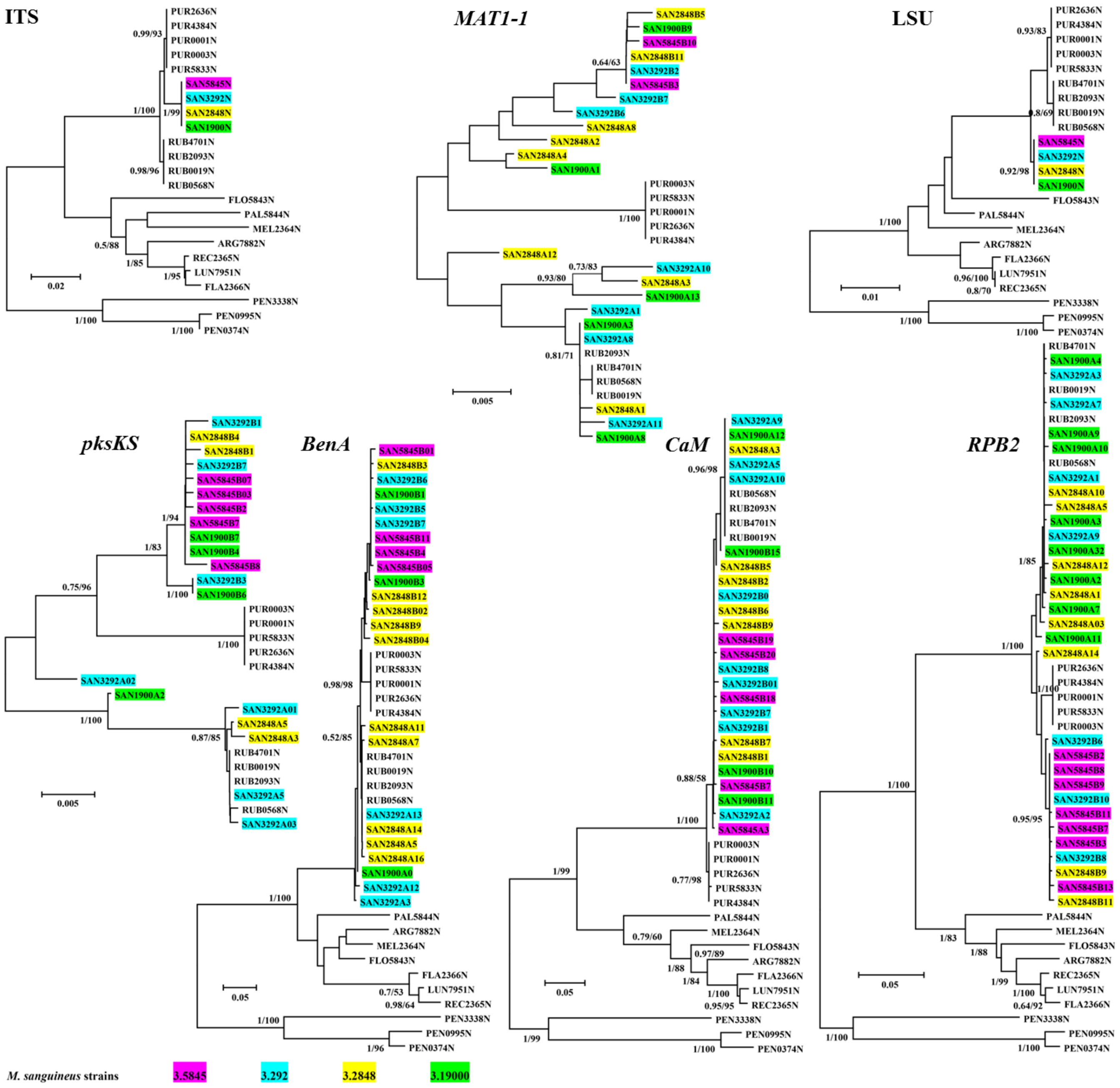

Figure S1. Single gene phylogenetic trees of the ITS, LSU, *BenA*, *CaM*, *RPB2*, *pksKS* and *MAT1-1* gene regions of species from *Monascus*.

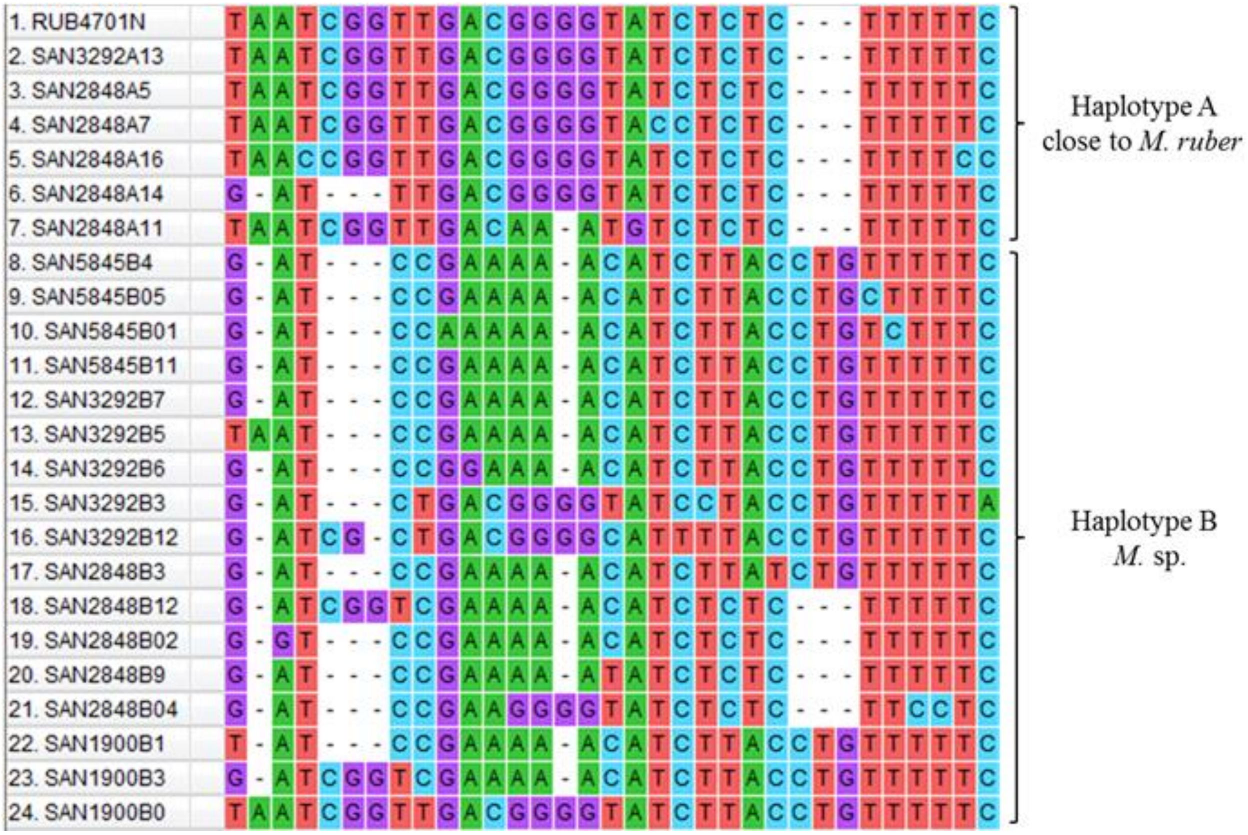

Figure S2. Two haplotypes of the *BenA* gene of four *M. sanguineus* strains (only inconsistent sequences are retained after alignment).

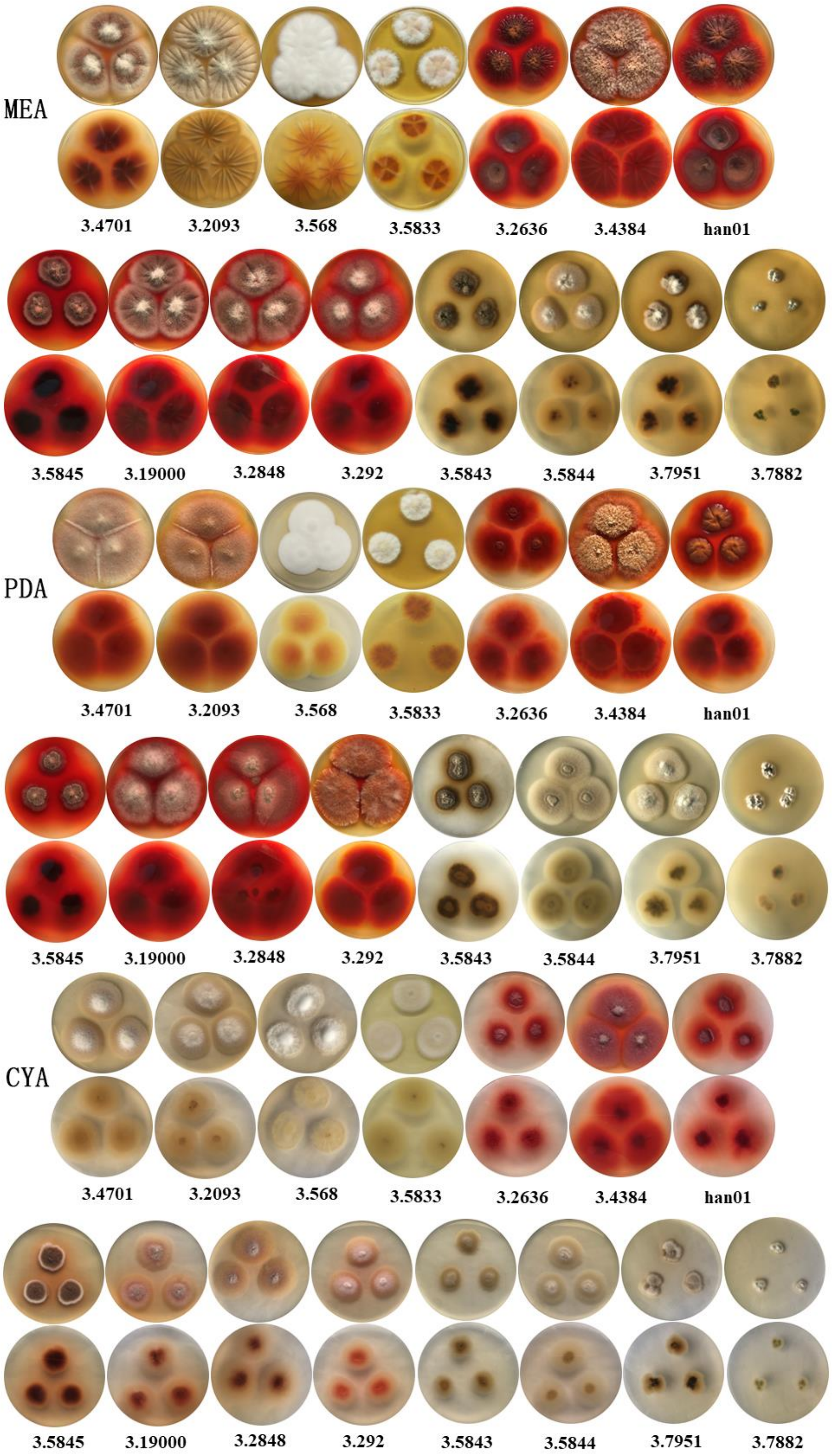

Figure S3. Cultural characters of 15 *Monascus* strains cultured on MEA, PDA, and CYA media at 30 °C for 7 days.

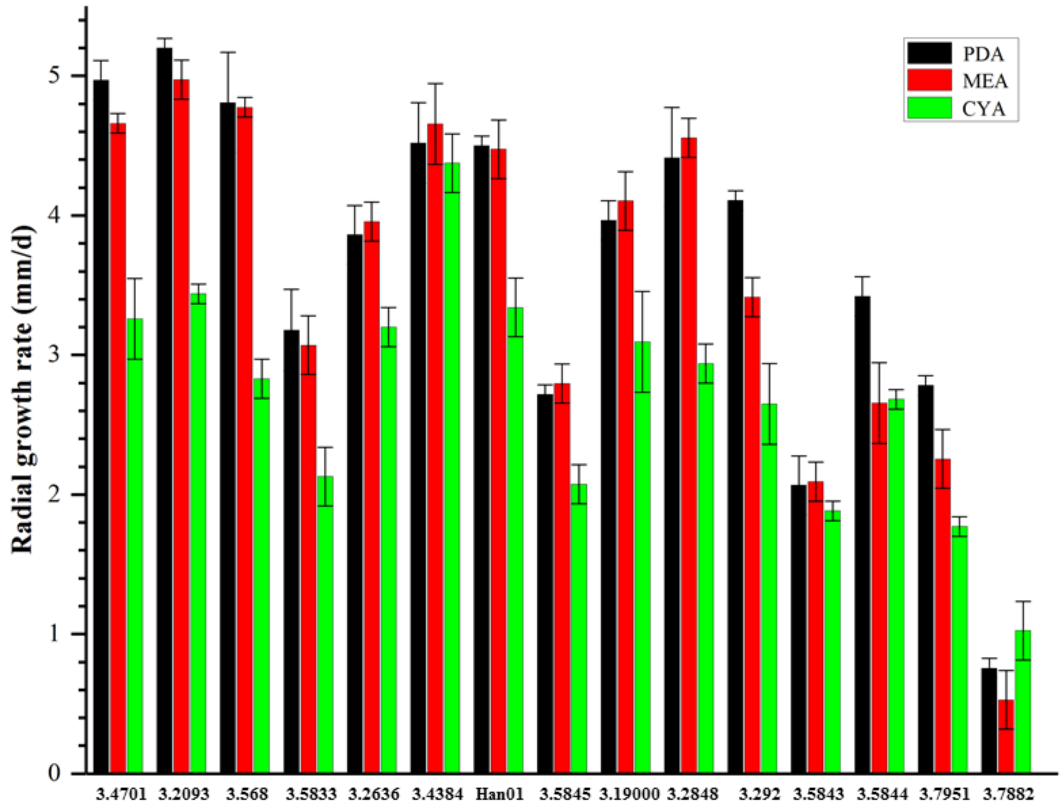

Figure S4. Mean growth rates (mm/d) of 15 *Monascus* strains cultured on PDA, MEA and CYA media at 30 °C for 7 days.
